# Life cycle assessment of MSW-to-biofuel conversion pathways: a comparative analysis

**DOI:** 10.1038/s41598-025-32082-y

**Published:** 2026-02-28

**Authors:** Rahul S. Raj, Siddharth Jain, Amit Kumar Sharma, Varun Pratap Singh

**Affiliations:** 1 Department of Mechanical Engineering, UPES, Dehradun-2, 48007 India; 2 Engines and Biofuels Research Laboratory, Department of R&D, College of Engineering Studies, UPES, Dehradun-2, 48007 India; 3 Solar Thermal Energy Research Group, Mechanical and Mechatronics Engineering Department, Stellenbosch University, Stellenbosch, 7600 South Africa

**Keywords:** Thermochemical conversion, Municipal solid waste, Life cycle assessment, Global warming potential, Waste to energy, Energy science and technology, Engineering, Environmental sciences

## Abstract

Rapidly increasing municipal solid waste (MSW) generation, reaching 160,039 tonnes per day in India, and the environmental burdens of conventional disposal highlight the need for efficient waste-to-biofuel solutions. This study conducts a comparative Life Cycle Assessment (LCA) of seven MSW-to-biofuel pathways: open landfilling, landfill gas recovery, incineration, torrefaction, gasification, hydrothermal carbonization, and integrated gasification. Using a functional unit of 1 tonne of MSW, the assessment quantifies environmental impacts across five midpoint categories (GWP, SOD, FEP, LU, WC) following ISO 14040/44 guidelines. The methodology integrates experimental MSW characterization, national waste statistics, and ± 10% sensitivity analysis to address uncertainties in methane capture, energy recovery, and grid displacement. Results show substantial differences across pathways, with integrated gasification (MIG) emerging as the most sustainable option, achieving an avoided GWP of − 1095 kg CO_2_ eq, water savings of − 1125.61 m^3^, and the lowest land-use requirement (− 32.39 m^2^·a). Material Flow Analysis further validates MIG’s superior mass-energy conversion when combined with recycling. The study’s novelty lies in its first holistic comparison of seven thermochemical and conventional MSW pathways tailored to India, integrating LCA and MFA evidence. the findings support prioritizing advanced thermochemical routes, particularly MIG, for climate-resilient, resource-efficient, and circular MSW management.

## Introduction

Municipal Solid Waste (MSW) management is a critical challenge for urban sustainability, particularly amid rapid population growth, industrialization, and economic development^1^. Globally, MSW generation is increasing at an alarming rate, as reported by Valavanidis^2^, with estimates suggesting that it will reach 3.4 billion tonnes annually by 2050 if current trends persist. Traditional waste management strategies, such as landfilling and open dumping, are associated with severe environmental impacts, including Greenhouse Gas (GHG) emissions, groundwater contamination, and land degradation^3^. The MSW generation worldwide is expected to rise to 3.4 billion tonnes by 2050 if current trends continue, as shown in Table 1. Improper waste management contributes significantly to environmental pollution, public health concerns, and climate change, with landfills alone accounting for nearly 11% of global methane emissions as reported by wang et al.^4^, and other researchers^5,6^. The increasing volume and complexity of waste require adopting efficient, sustainable waste management strategies that minimize environmental impact while maximizing resource recovery.

At the same time, the energy crisis and the urgent need for sustainable alternatives to fossil fuels have intensified interest in waste-to-energy (WtE) technologies. Traditionally, MSW has been managed through landfilling and open dumping, which remain the dominant disposal methods in many countries due to their low operational costs . However, these methods pose serious environmental risks, including GHG, leachate contamination, and land degradation. However, to address the environmental concerns associated with conventional disposal, various WtE and material recovery processes have been developed, offering alternative pathways for waste utilization. Among them, incineration, gasification, pyrolysis, and hydrothermal carbonization (HTC) have gained attention as effective strategies for converting MSW into energy-dense biofuels and valuable byproducts^7,8^. These technologies not only reduce reliance on landfills but also contribute to renewable energy generation.

**Table 1:** Global MSW statistics, composition, and key management challenges.

Aspect	Global details/values	Indian context	Environmental impact	Remarks	References
Annual MSW	~ 2.01 Bt/y to ~ 3.40 Bt/y (2050)	~ 62 Mt/y; fast-growing	Increases GHGs, resource stress	Driven by urbanization, pop. growth	^9^
Composition	Organic (50–60%), Paper, Plastics, etc	Higher organics, low recyclables	Methane (organics), persistence (plastics)	Impacts tech choice; increases biogas in India	^10,11^
Practices	Incineration, recycling increases; landfill decreases	Mostly open dumping, limited segregation	Methane, leachate, poor recovery	Infra & awareness gaps persist	^12^
Challenges	Emissions, leachate, low circularity	Overcrowded dumpsites, weak recycling	Soil, water, air degradation	Socio-economic factors	^13^
Policy	Strong regulatory frameworks in developed nations	SWM Rules 2016, EPR framework	Potential for major impact	Implementation bottlenecks remain	^14,15^

*Bt = Billion tonnes; Mt = Million tonnes.

Thermochemical processes such as torrefaction, gasification, and HTC convert MSW into syngas, biochar, and bio-oil, which can be used for power generation and industrial applications^16,17^. Torrefaction, a mild heating process, enhances the properties of solid biofuels for co-firing in existing energy infrastructure^7,18^. HTC operates in a water-rich environment, as reported by Libra et al.^16^, producing hydrochar with high carbon content, suitable for biofuel applications.

Despite advancements in waste-to-biofuel conversion technologies, there remains a significant gap in understanding their overall environmental implications. Life Cycle Assisment (LCA) provides a systematic framework, based on ISO 14040/44, to quantify the environmental impacts of these technologies by evaluating emissions, resource consumption, and energy efficiency across different stages of the process^12^. Previous studies by Haitao et al.^19^, Sarkar et al.^6^, and Kashem et al.^12^ have analysed individual conversion pathways; a comprehensive comparative LCA of multiple MSW-to-biofuel scenarios is needed to assess their long-term viability. This study aims to bridge this gap by evaluating and comparing the environmental trade-offs associated with different waste treatment technologies.

While previous studies have examined individual MSW conversion pathways^8,20,21^, this study represents the first comprehensive comparative LCA of seven distinct MSW-to-biofuel scenarios tailored explicitly to Indian waste composition and operational conditions. The novelty lies in three key aspects: (i) integration of primary experimental data from Dehradun MSW samples with nationally representative waste generation statistics (160,039 tpd), bridging the gap between laboratory-scale characterization and national-level environmental assessment; (ii) systematic comparison of emerging technologies (torrefaction, hydrothermal carbonization, integrated gasification) alongside conventional methods (landfilling, incineration) under standardized functional units and system boundaries; and (iii) validation through Material Flow Analysis from our previous work^11^, demonstrating convergence between material balance optimization and environmental performance assessment. Unlike prior Indian LCA studies focusing on single technologies^22–24^ or limited impact categories^25^, this work evaluates five midpoint indicators (GWP, SOD, FEP, LU, WC) across all pathways, enabling holistic technology prioritization for India’s 62 Mt/year MSW challenge.

This study evaluates seven MSW-to-biofuel scenarios, each representing a distinct waste management strategy with varying degrees of energy recovery and environmental impact. The first scenario illustrates the baseline case, where waste is disposed of in landfills without energy recovery, resulting in uncontrolled methane emissions and leachate formation. The second scenario considers landfilling with landfill gas recovery, in which methane emissions are captured and used for electricity generation. The third scenario examines incineration with energy recovery, in which MSW is combusted at high temperatures to generate electricity while managing residual ash. The fourth scenario focuses on torrefaction, which converts MSW into torrefied biomass, a solid biofuel suitable for co-firing in power plants. The fifth scenario explores gasification, a high-temperature thermochemical process that converts MSW into syngas and biochar. The sixth scenario evaluates HTC, a water-based thermochemical process that transforms MSW into hydrochar, a potential biofuel with high carbon content. The seventh scenario examines two-stage gasification with first-stage torrefaction, followed by gasification of MSW into syngas.

By applying an LCA framework, this study aims to compare the environmental impacts of conventional and advanced MSW-to-biofuel pathways. It seeks to identify key environmental hotspots within each conversion process and to assess the GWP and energy recovery. The findings of this research will help prioritize low-emission, high-efficiency waste-to-biofuel technologies, with a high Energy Return on Investment (EROI) scenario contributing to climate change mitigation, renewable energy generation, and circular economy goals.

## Materials and methods

### Goal and scope

This study employs an LCA approach to evaluate and compare the environmental impacts of different MSW-to-biofuel conversion pathways.

**Fig. 1:**
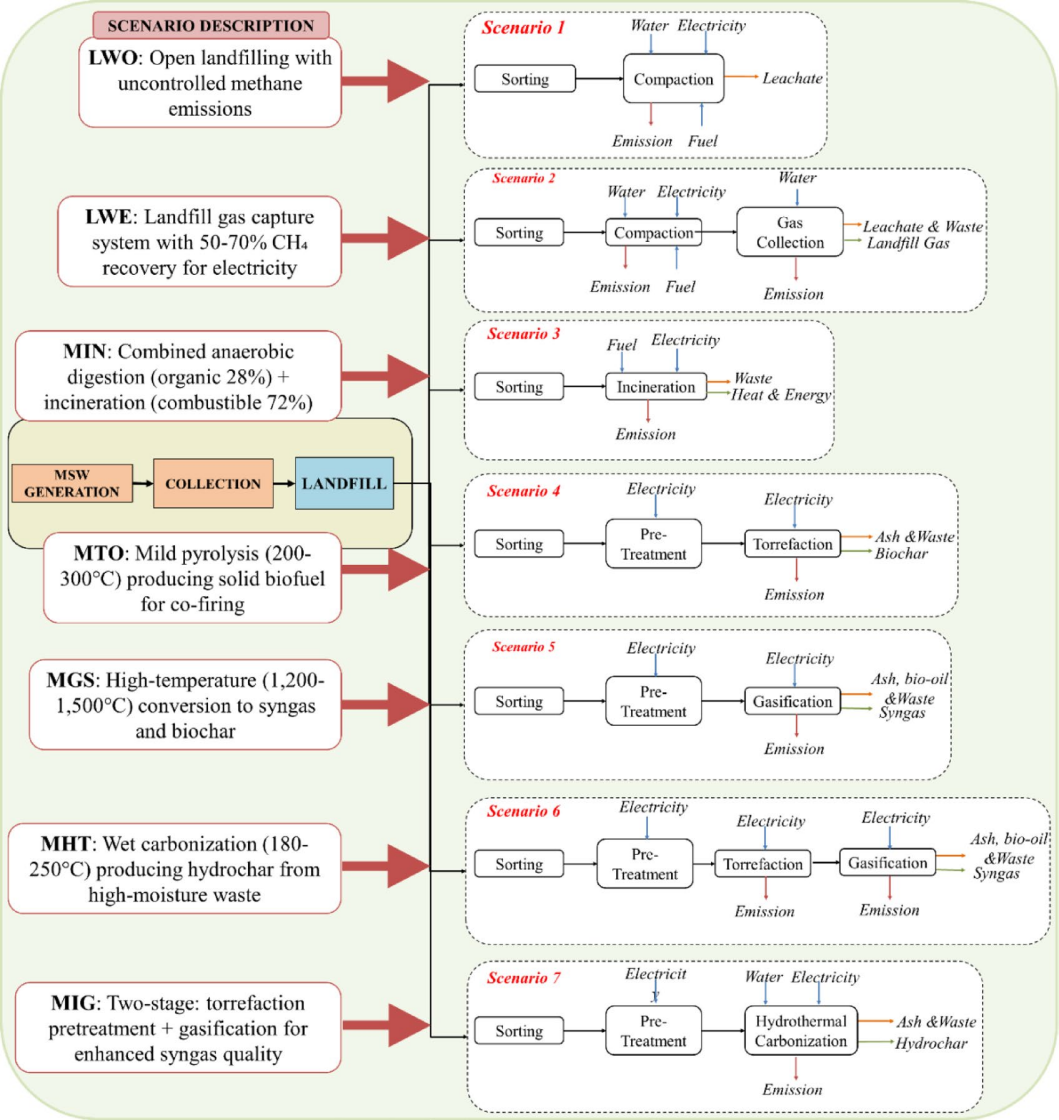
LCA framework based on ISO 14040/44 standards, showing the stages from goal and scope definition to impact assessment and interpretation for all scenarios.

The LCA framework follows the ISO 14040 and ISO 14044 standards, ensuring a systematic, standardised evaluation of the environmental implications of each waste treatment scenario, as shown in Fig. 1^20,21,26^. The assessment is conducted using four key phases: goal and scope definition, LCI, LCIA, and interpretation. The primary objective of this LCA is to quantify the environmental burdens associated with each waste-to-biofuel process and identify the most sustainable waste management strategies.

### Sampling and site selection

**Fig. 2:**
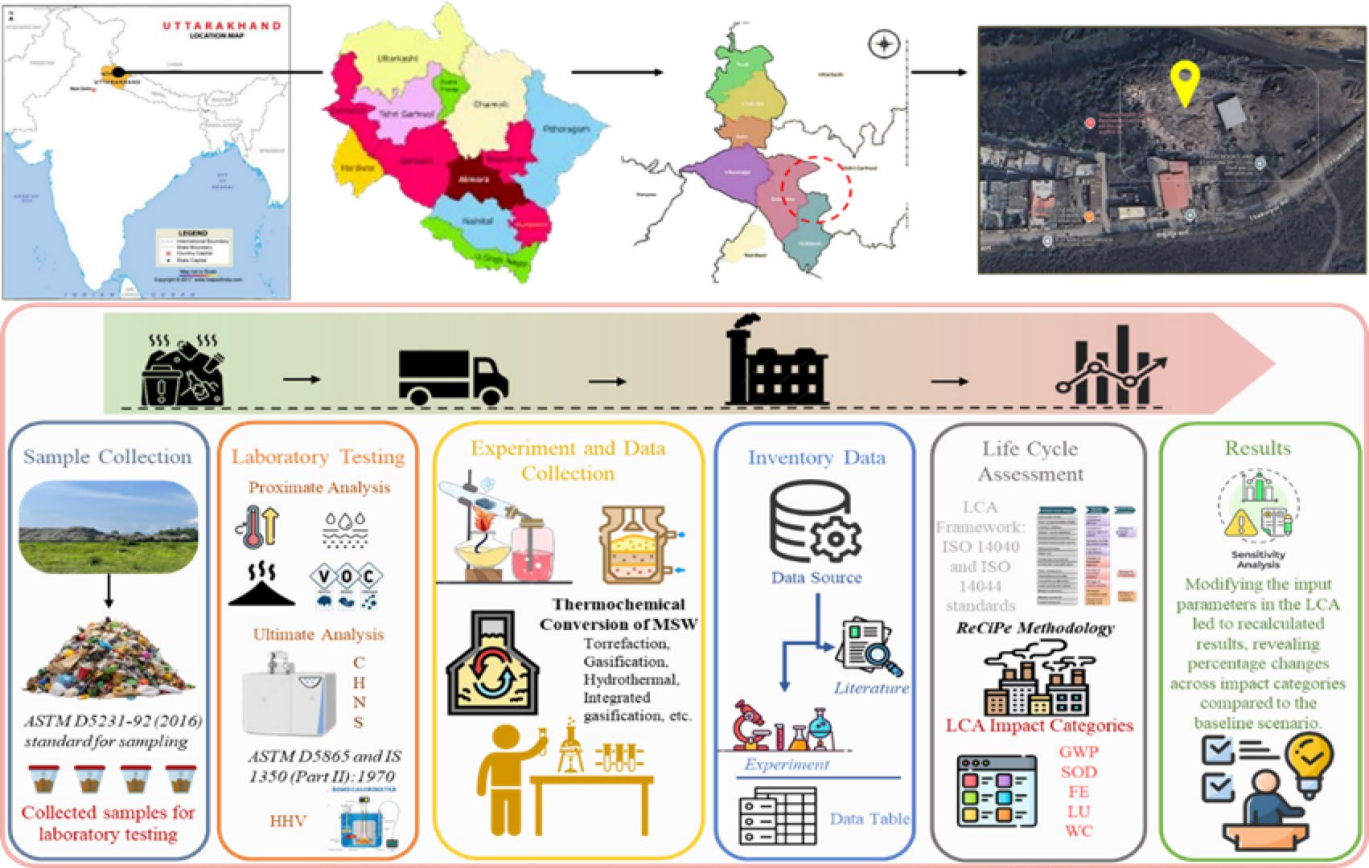
Study scope steps and area map (Prem Nagar dump yard, Dehradun City).

MSW samples were collected from the Prem Nagar dumping site in Dehradun, India, as shown in Fig. 2, using a stratified random sampling method consistent with the American Society for Testing and Materials (ASTM) D5231-92 (2016) to obtain a representative composition of the mixed waste stream. The collected samples were transported in sealed, opaque, high-density polyethene containers to prevent exposure to sunlight and to minimise material degradation. At the laboratory, the samples were manually sorted to remove any unwanted materials. The sorted sample was characterized for its key thermal and physical properties, including moisture content, proximate composition, and calorific value.

Moisture content was analyzed by oven drying at 105 ± 2 °C until constant weight, while proximate analysis followed ASTM D3172 and IS 1350 (Part I):1984 to determine volatile matter, ash content, and fixed carbon. The HHV of each fraction was determined in accordance with ASTM D5865 and IS 1350 (Part II):1970, using data from prior experimental trials. These experimentally obtained values were subsequently scaled and integrated into the LCI to model the environmental performance of MSW treatment systems on a functional-unit basis^27^.

### Functional unit

A functional unit of 1 metric tonne of MSW processed is defined to standardize comparisons across all scenarios^9^. This unit serves as a consistent reference point, as reported by many researchers, enabling equivalent assessments of various conversion pathways by accounting for differences in waste composition, energy recovery potential, and byproduct generation^23,24^. The selection of one metric tonne as the functional unit is justified by three considerations: (i) policy alignment—India’s Solid Waste Management Rules 2016 and municipal waste reporting standards (Central Pollution Control Board Annual Reports) consistently use tonne-based metrics^13,28^; (ii) operational scalability—facility capacities are typically specified in tonnes per day (tpd), enabling direct translation of LCA results to plant-level performance (e.g., a 500 tpd facility processing 160,039 tpd nationally would require ~ 320 plants); and (iii) comparative consistency—established waste LCA literature^29–31^ predominantly employs mass-based functional units, facilitating benchmarking against international studies. This unit enables equivalent assessments across pathways with disparate waste composition tolerances and energy recovery potentials^32^.

The system boundaries adopt a gate-to-gate perspective for the treatment facility, encompassing all processes from MSW arrival through pre-treatment, conversion, energy recovery, and residue management. Transportation from collection points to the facility (an average distance of 40 km, documented in Table 3 is included as a foreground process due to its direct operational relevance. Avoided environmental burdens from grid electricity displacement and material substitution (e.g., fertilizer, biochar) are credited^21^ using the ISO 14044 system expansion methodology^21^, with life-cycle emission factors consistent with ecoinvent v3.8^25^ and established waste LCA practice^29,33,34^. Upstream processes (capital equipment manufacturing, infrastructure construction, fuel production chains) are excluded based on < 2% contribution thresholds demonstrated in prior waste-to-energy LCA studies^11,35,36^. This boundary choice prioritizes operational-phase impacts most relevant to technology selection while maintaining consistency with comparative waste LCA frameworks^24,37^.

Key assumptions in the analysis include the use of region-specific MSW composition data, conversion efficiencies informed by both literature and our laboratory experimental results, and the incorporation of the regional grid electricity mix to estimate energy inputs and offsets^21,38^. Uncertainties in these assumptions are addressed through sensitivity analysis^21^. The LCI data are drawn from a combination of primary data collected through experimental studies, and secondary sources including peer-reviewed literature by Dangi et al.^28^, Lin et al.^19^, and others^39–41^. Environmental impacts are evaluated using multiple impact categories, including GHG emissions, energy balance, ozone depletion potential, eutrophication potential, land-use potential, and water quality potential^42^. These are quantified using the Life Cycle Inventory Analysis (LCIA) methodology, ReCiPe, ensuring comprehensive environmental profiling of each scenario^39,43^.

The selection of impact categories was based on their direct relevance to MSW treatment systems and regional environmental priorities. GWP was prioritized due to India’s commitment to climate action under the Paris Agreement and the significant methane emissions from landfills^44,45^. SOD was included to capture potential halogenated compound releases from mixed waste streams^24^. FEP addresses nutrient discharge from leachate, a critical concern in regions with limited wastewater treatment infrastructure^29^. LU reflects spatial pressure in densely populated urban areas where land scarcity is acute^46,47^. WC evaluates freshwater demand, particularly relevant in water-stressed regions of India^48^.

While toxicity impacts (human toxicity, ecotoxicity) and particulate matter formation are acknowledged as relevant, particularly for thermal treatment pathways, their exclusion from this study is justified by several factors. First, modern incineration facilities in India are required to comply with stringent emission standards (CO < 100 mg/Nm^3^, NOx < 400 mg/Nm^3^, dioxins < 0.1 ng TEQ/Nm^3^) as per Central Pollution Control Board guidelines^23,49^. Second, the primary objective of this study is a comparative assessment of energy recovery potential and climate impact mitigation, which are best represented by the selected categories. Third, data availability and uncertainty for toxicity characterization factors in the Indian context remain limited^13,40,50^. Future studies should incorporate human toxicity potential and freshwater ecotoxicity using region-specific characterization factors to provide a more comprehensive environmental profile, particularly when evaluating specific facility designs with detailed emission control specifications.

### Scenario development

**Fig. 3:**
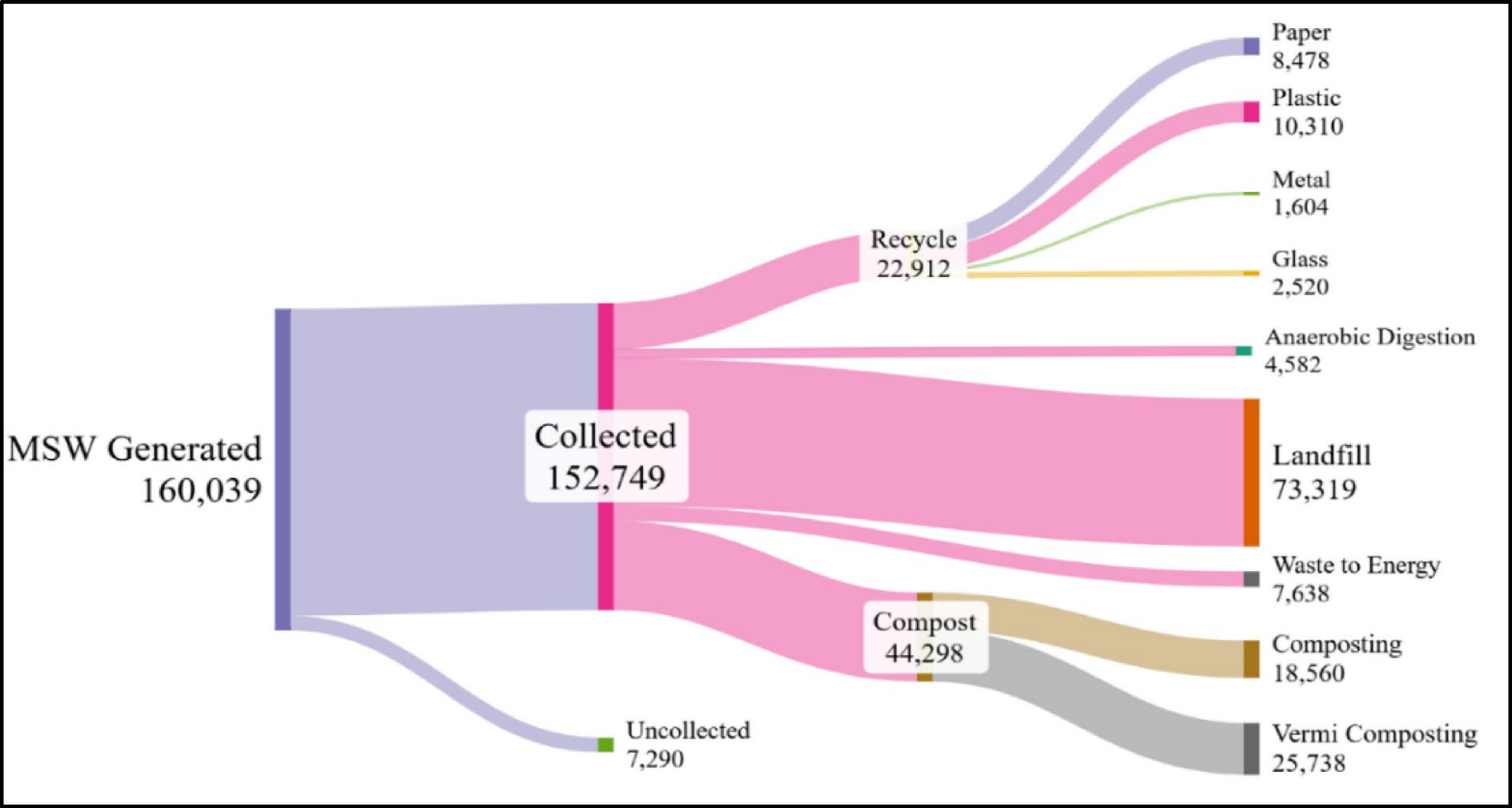
Sankey diagram showing current MSW management routes in India.

India’s MSW landscape is characterized by a complex, heterogeneous mix of materials, including organic matter, paper, plastics, metals, and inert substances^51–53^. This complexity necessitates preprocessing steps such as sorting, drying, and homogenization to enable efficient conversion. The current waste management system in India, illustrated through the Sankey diagram in Fig. 3, shows a total MSW generation of 160,039 tonnes per day, of which approximately 152,749 tpd is collected^8^. From this collected waste, 22,912 tpd is directed to recycling, 4582 tpd to anaerobic digestion, 18,560 tpd to composting, 25,738 tpd to vermi-composting, 7638 tpd to waste-to-energy (WtE) plants, and the most significant fraction of 73,319 tpd is still being disposed of in landfills, often without any energy recovery^54,55^.

This distribution highlights a significant reliance on conventional disposal and low-efficiency treatment methods, especially landfilling. The dominance of landfills, despite their environmental drawbacks such as leachate contamination, methane emissions, and land-use burdens, underscores the urgent need to evaluate alternative, more sustainable waste-to-energy conversion routes. Although India has initiated WtE projects, their scale and efficiency remain limited relative to the country’s total waste burden, and treatment methods such as anaerobic digestion and composting have yet to achieve optimal operational coverage^49^. To address these challenges and identify more efficient, environmentally sound pathways, this study evaluates seven distinct scenarios that combine conventional, intermediate, and advanced MSW-to-biofuel conversion technologies.

#### Conventional landfilling approaches

Landfill without energy recovery (LWO) remains the most basic method, involving waste deposition without energy capture. It has low costs but poses serious environmental issues, including methane emissions and^56^. Landfill with energy recovery (LWE) integrates systems to capture landfill methane for energy use. Though it reduces emissions, only 50–70% of methane is typically recovered, and system costs are high^57^.

In LWO, no energy is recovered, so no emissions are avoided. In LWE, captured methane (approximately 60% capture efficiency^57^) is combusted in gas engines, generating ~ 220 kWh of electricity per tonne MSW processed^56,58^. This displaces Indian grid electricity (0.82 kg CO_2_ eq./kWh), resulting in avoided emissions of 155.80 kg CO_2_ eq. per tonne. The remaining 40% of methane escapes as fugitive emissions, contributing significantly to the net GWP. Leachate is collected and treated in engineered systems, though treatment processes consume additional energy (15–20 kWh/tonne)^12^. The residual waste mass remains permanently in the landfill, occupying land for 50 + years post-closure^57^.

#### Thermal conversion technologies

Incineration burns MSW above 800 °C, reducing volume by up to 90% and generating electricity or heat. While modern plants have advanced flue gas treatment, concerns persist over toxic emissions and ash management^59^. Torrefaction mildly heats MSW (200–300 °C) under low-oxygen conditions to produce an energy-dense, hydrophobic fuel. It enhances grindability and calorific value, but sustainability depends on energy recovery and emission controls^60^. Gasification converts organic MSW into syngas via high-temperature (1200–1500 °C) reactions with limited oxygen. When optimized, it enables high energy recovery, biochar production, and low emissions, but is sensitive to feedstock and process control^61,62^. Integrated Gasification couples torrefaction and gasification to enhance feedstock quality and syngas purity, improving system stability and conversion efficiency while reducing emissions^63^.

*MSW incineration (MIN)*: The organic fraction (28%) undergoes anaerobic digestion, producing biogas (60% CH_4_ content), which generates approximately 150 kWh of electricity^64,65^. The combustible fraction (72%) is incinerated at 850–1000 °C, producing 650–800 kWh of electricity^65^. Total electricity output is ~ 950 kWh/tonne, with self-consumption of 300–350 kWh for sorting, heating digesters, and pollution control, resulting in net export of 450–600 kWh^22^. Digestate from anaerobic digestion (200–250 kg/tonne) is used as soil amendment, displacing 10–15 kg synthetic fertilizer N-equivalent^66^. Bottom ash (15–20% of input mass) requires disposal in secure landfills^67^. Avoided emissions include grid electricity displacement (369 kg CO_2_ eq.) and fertilizer substitution (38 kg CO_2_ eq.), totalling approximately 407 kg CO_2_ eq. per tonne^25^.

*MSW Torrefaction (MTO)*: Torrefaction produces energy-dense solid biofuel (torrefied biomass) with 70% mass yield and enhanced calorific value (HHV: 20–23 MJ/kg vs. 15–17 MJ/kg for raw MSW)^7,60^. This material is suitable for co-firing in existing coal power plants or gasification. When combusted, it generates approximately 1350–1500 kWh of electricity per tonne of MSW processed^46,61^. Self-consumption (drying, torrefaction heating) is 150–200 kWh/tonne, yielding a net export of 1200–1350 kWh^68^. The process produces 20–30% gaseous/condensable byproducts that can be recycled for process heat. Ash content is concentrated in the torrefied product (8–10%) but remains manageable^61^. Avoided emissions from grid displacement: approximately 1050 kg CO_2_ eq. per tonne**.**

*MSW Gasification (MGS)*: Gasification converts MSW into syngas (CO, H_2_, CH_4_, CO_2_) and solid biochar (15–20% yield)^24,69^. The syngas LHV ranges from 4 to 6 MJ/Nm^3^, depending on the gasifying agent (air vs. oxygen/steam)^70^. Energy conversion via gas engines or turbines produces 1400–1600 kWh of electricity per tonne of MSW, with 100–120 kWh ofelf-consumption for drying, shredding, and gas cleaning^29,69^. Net electricity export: approximately 1480 kWh. Biochar (180 kg/tonne) serves as a soil amendment with carbon sequestration potential (50% stable carbon over 100 + years), providing additional avoided burden of 90 kg CO_2_ eq.^29^. Slag/ash (8–10%) is inert and suitable for construction material reuse^69^. Total avoided emissions: 1303 kg CO_2_ eq. per tonne, comprising grid displacement (1213 kg) and biochar credit (90 kg).

*MSW Integrated gasification (MIG)*: This two-stage process first torrefies MSW (200–300 °C) to improve feedstock properties (reduced moisture, enhanced grindability, increased energy density), then gasifies the torrefied material at higher temperatures (1200–1500 °C)^71^. The integration yields higher-quality syngas (H_2_ content 20–25% vs. 12–15% in single-stage MGS) with improved LHV (6–7 MJ/Nm^3^)^71^. Electricity generation reaches 1700–1800 kWh/tonne, with self-consumption of 180–200 kWh, yielding a net export of 1500–1600 kWh^71^. Combined heat and power (CHP) configurations allow the recovery of process heat (300–400 kWh of thermal energy) for district heating or industrial applications^72,73^. Biochar yield is lower (10–15%) but of higher quality due to controlled two-stage carbonization^73^. Total avoided emissions: 1475 kg CO_2_ eq., including grid electricity displacement (1312 kg), biochar sequestration (53 kg), and heat recovery credits (110 kg)—resulting in the most favourable net GWP among all scenarios.

Table 2 provides a qualitative comparison between conventional landfilling (representing the baseline LWO scenario) and integrated gasification (MIG scenario), highlighting the fundamental technological and environmental trade-offs inherent in MSW management decision-making. While open dumping requires minimal capital investment and technical expertise, it imposes substantial ecological externalities, including uncontrolled GHG emissions, leachate contamination, and long-term land occupation^29,67^. Advanced thermochemical systems like MIG demand significant upfront investment and operational sophistication but offer transformative environmental benefits through energy recovery, reduced waste volumes, and controlled emissions^71^. This comparison frames the subsequent LCA results, which quantify these qualitative differences across five environmental impact categories.

**Table 2:** Comparative overview of conventional landfilling versus the best-performing advanced conversion technology (MIG).

Characteristic	Open dumping (Landfill)	Integrated gasification (MIG)
Energy Recovery	None	High (1600 kWh net/tonne)
Climate Impact	Very High (uncontrolled CH_4_)	Beneficial (net negative GWP)
GHG Emissions	High (736 kg CH_4_/tonne)	Low (controlled, mostly biogenic CO_2_)
Toxic Emissions	Uncontrolled	Controlled (advanced flue gas treatment)
Residual Mass	100% remains	Minimal (5–8% inert ash)
Economic Cost	Low direct cost (high externalities)	High capital investment
Leachate Management	Uncontrolled contamination	Minimal (dry process)
Infrastructure Required	Minimal	Advanced technology (gasification reactors, gas cleaning)
Land Requirement	High (68 m^2^·a/tonne)	Low (− 32.39 m^2^·a/tonne net)
Water Consumption	Minimal direct use	Low with high savings (− 1125.61 m^3^/tonne net)
Technology Maturity	Well-established	Emerging (limited commercial deployment)
Operational Complexity	Simple	Complex (requires skilled operation)

#### Emerging hydrothermal treatment

HTC treats wet organic MSW (180–250 °C, 2–10 MPa) in water to form hydrochar without prior drying. It is suited for high-moisture waste, such as food scraps and sewage sludge, though liquid byproducts require further treatment. Its viability hinges on process optimization and effluent management^5,74^.

HTC produces hydrochar (30–40% mass yield) with enhanced carbon content (50–60% vs. 35–45% in raw MSW) and energy density (HHV: 18–22 MJ/kg)^5,19^. The process operates in an aqueous phase, eliminating the need to pre-dry high-moisture waste streams^74^. Process water contains dissolved organics (COD: 20–40 g/L) and nutrients (N, P), requiring treatment before discharge or recirculation^5^. When treated via anaerobic digestion, this water yields additional biogas (10–15% energy bonus)^5^. Hydrochar combustion generates 1200–1400 kWh of electricity per tonne of MSW processed. However, the process is energy-intensive: heating water to 180–250 °C and maintaining pressure (2–6 MPa) consumes 400–500 kWh, plus water treatment requires 80–100 kWh^5,74^. Net electricity export: approximately 700–900 kWh^74^. Process water recirculation (70–80%) significantly reduces freshwater consumption, providing water savings of 800–950 m^3^ per tonne MSW—the second highest among thermal pathways^61^. Avoided emissions total approximately 1044 kg CO_2_ eq., primarily from grid electricity displacement (722 kg) and water resource conservation credits (322 kg)^29^. The hydrochar can also serve as a solid fuel, an activated carbon precursor, or soil conditioner, though its high ash content (15–20%) limits some applications^39,74^.

### LCI data

LCI data^25,40,43^are gathered from multiple sources, including experimental studies from the existing literature^25,30,32^. Process-specific inputs and outputs, including material and energy flows, emissions, and waste residues, are systematically recorded for each scenario, as given in Table 3. Site-specific data is incorporated where available, particularly for waste composition, landfill operations, and waste-to-energy facility parameters. Uncertainties in the LCI data are addressed through sensitivity analyses to ensure robust results.

**Table 3:** General Inventory data for LCIA^4,25,75–79^.

Parameters	Value	
Distance to and from (km)	40	
Diesel Consumption (litres/tonne)	3	
Input/ Output	Indicator Parameters	Unit and Interpretation
Input	MSW	t, Daily MSW generated in the area
	Diesel	Diesel consumption in transportation and other processes
	Electricity	kwh, Self-electricity consumption
Output	Electricity	kwh, Net electricity output
	Materials	Products (solid, Liquid, Gas)
GHG emissions	CO_2_^eq^	kg, Total GHG emissions are calculated by CO_2_ equivalent
Gasification	CO_2_	kg, major sources of emissions
Transportation diesel	CO_2_	kg, Diesel emissions during transportation
	CH_4_	kg
	N_2_O	kg
Auxiliary fuel	CO_2_	Kg, Auxiliary fuel emissions of gasification
	CH_4_	kg
	N_2_O	kg
Pollutants	Emissions kg/L of diesel	Emissions kg/tonne of MSW
Carbon monoxide	0.012	0.036
Hydrocarbon	0.00175	0.00525
Carbon dioxide	2.66	7.99
Nitrogen oxides	0.00236	0.0071
Particulate matter, PM 2.5	0.00062	0.00186
Impacts associated with open dumping for the base scenario		Value
Air Environment (Pollutants)		Emissions (kg/tonne)
Hydrocarbons		0.00475
Hydrogen Sulfide		0.312
PM (2.5–10)		0.0,00,204
Ammonia		6.89
Methane		736
Nitrogen Oxides		0.00129
Sulphur dioxide		0.00475
Carbon dioxide		630
Carbon monoxide		0.00276
Water Environment (Pollutants)		Emissions (kg/tonne)
Arsenic		9.07 × 10^–7^
Cadmium		3.89 × 10^–7^
Chromium		1.44 × 10^–6^
Copper		3.10 × 10^–6^
Lead		2.74 × 10^–7^
Nickel		5.11 × 10^–7^
Zinc		6.99 × 10^–8^
Nitrogen, total		0.165
Phosphorous, total		0.119
Soil Environment (Pollutants)		Emissions (kg/tonne)
Calcium		0.586
Iron		1.74 × 10^–2^
Magnesium		0.592
Manganese		0.102
Nickel		5.40 × 10^–4^
Nitrogen		5.15 × 10^–2^
Phosphate		4.53 × 10^–2^
Potassium		0.163
Sodium		0.102
Zinc		2.25 × 10^–3^

All impact calculations performed using ReCiPe 2016 v1.1 (H) midpoint characterization factors. All emission factors at standard conditions (273 K, 101.3 kPa).

Given the complexities of MSW management, it is crucial to account not only for conversion technologies but also for the environmental implications of each pathway. The integration of LCA methodologies provides a comprehensive framework for evaluating these impacts, particularly with respect to energy consumption and GHG emissions throughout the biofuel production process. The environmental impact categories considered in this study include GWP, SOD, FE, LU, and WC. GHG emissions are a critical focus, given MSW management’s significant contribution to methane and carbon dioxide emissions^9^. The energy balance assessment accounts for both the energy consumed and produced in each scenario, ensuring a net energy analysis. The LCIA is performed using the established ReCiPe impact assessment methodology^9,71^. The LCIA translates LCI data into quantifiable environmental impacts, allowing for a comparative evaluation of each scenario. Sensitivity analysis is conducted to assess the influence of key parameters, such as energy efficiency, waste composition, and transportation distances, on overall results^80,81^.

The quality and reliability of LCI data were ensured through systematic source selection and validation. Primary experimental data (MSW characterization, conversion trials) were obtained using standardized protocols (ASTM D5231-92, D3172, D5865), with documented measurement uncertainties < 8% for replicate samples. Secondary data were prioritized from: (i) peer-reviewed literature published within the last 5 years for technology-specific parameters^11,14^, (ii) official government sources for national waste statistics and grid emission factors^13,42,49^, and (iii) established LCA databases (ecoinvent v3.8) for background processes^42^. Geographic representativeness was maintained by using India-specific data wherever available (MSW composition^9,11,53,82^, grid mix^42^, landfill conditions^5,58,83,84^), with international data adapted only for emerging technologies (HTC, MIG) where Indian commercial-scale operations remain limited. Parameter-specific uncertainties, ranging from ± 5% for well-established factors (transportation emissions) to ± 20% for site-variable processes (landfill methane capture efficiency), are addressed through sensitivity analysis (Section "Sensitivity analysis").

### Life cycle impact assessment

In the present study, LCIA was employed to evaluate the potential environmental impacts of different MSW management scenarios, with a particular focus on integrating an advanced thermochemical conversion pathway. Impact categories relevant to waste treatment were selected based on their significance in prior literature and international standards^24,47,69,85,86^. The ReCiPe 2016 v1.1 midpoint (H) method was employed for impact characterization, using the hierarchical perspective with a 100-year timeframe as recommended for policy-relevant assessments^52,53^. GWP, measured in kg CO_2_-equivalent, quantifies the cumulative climate impact from greenhouse gas emissions generated during feedstock handling, conversion processes, and energy recovery or residue management^45,87^. SOD, expressed in kg of Chloro Fluoro Carbon (CFC-11)-equivalent, accounts for the potential release of halogenated compounds, particularly from high-temperature treatments involving mixed or poorly segregated waste fractions^29,88^. FEP, reported in kg P-equivalent, reflects the discharge of nutrient-rich leachates and effluents that can disturb aquatic ecosystems, with variations across scenarios depending on the degree of leachate control and nutrient recovery^89^. Land Use (LU), calculated in m^2^·a crop-equivalents, evaluates the spatial footprint required for infrastructure, residue disposal, and supporting facilities, serving as an indicator of potential land pressure and ecosystem disruption^90^. Water Consumption (WC), measured in m^3^ per tonne of MSW, captures the total freshwater demand throughout the system, including pre-treatment, conversion, and pollution control stages, while accounting for savings from avoided conventional resource use. Emissions and resource flows derived from the LCI were classified into these categories and subsequently characterized using ReCiPe 2016.

The selection of these five impact categories was guided by ISO 14044 principles of relevance, measurability, and significance, with particular emphasis on MSW management priorities in the Indian context. GWP was prioritized due to India’s commitment to climate action under the Paris Agreement and the significant contribution of landfill methane emissions (11% of global CH_4_)^5,26^. SOD was included to capture potential releases of halogenated compounds from mixed waste streams, particularly relevant given limited source segregation in Indian municipalities^29,88^. FEP addresses nutrient discharge from leachate, a critical concern in regions with limited wastewater treatment infrastructure^89^. LU reflects spatial pressure in densely populated urban areas where land scarcity is acute and competing land uses (agriculture, housing, industry) create high opportunity costs^90^. WC evaluates freshwater demand, particularly relevant in water-stressed regions of India, where per capita water availability has declined 70% since 1950^90,91^. These categories align with India’s National Action Plan on Climate Change priorities and the Sustainable Development Goals (SDGs 6, 7, 11, 13), ensuring policy-relevant assessment.

### Sensitivity analysis

In this study, Sensitivity Analysis was applied to evaluate how variations in input parameters affect the LCIA results for the seven MSW-to-biofuel scenarios. Each of these scenarios represents a distinct waste management strategy, and sensitivity analysis is essential for assessing the robustness of the environmental impacts identified, particularly regarding energy recovery and GHG emissions^92^.

The ± 10% range was selected to reflect composite operational uncertainties rather than emission-factor uncertainties alone. While IPCC provides standardized GWP values for individual gases (CH_4_ = 28 kg CO_2_ eq., N_2_O = 265 kg CO_2_ eq. for 100-year timeframe), real-world MSW treatment systems exhibit process variability due to: (i) feedstock heterogeneity and seasonal composition changes^26,40^; (ii) operational parameter fluctuations in conversion efficiency^69^; (iii) regional grid electricity mix variations^28^; and (iv) measurement uncertainties in emission quantification^60^. The ± 10% range has been validated in prior MSW LCA studies as representative of these combined uncertainties^7,60^. To complement this analysis, scenario-specific sensitivity analyses of grid emission factors (0.74–0.90 kg CO_2_/kWh, reflecting India’s coal-dominated grid with increasing renewable penetration) were also conducted, which directly influence avoided burden calculations for all energy-recovery scenarios.

The relevance of sensitivity analysis lies in its ability to identify the parameters that most significantly affect each scenario’s outcomes. In scenarios such as LWE, small changes in gas capture efficiency can have a substantial impact on GWP. Similarly, for thermochemical processes such as gasification, torrefaction, and HTC, fluctuations in feedstock quality or process parameters can directly affect the system’s carbon footprint. Sensitivity analysis helps to pinpoint these critical factors, highlighting areas where more accurate data could improve the reliability of the LCA results. By conducting sensitivity analysis, this study ensures that data uncertainties do not unduly influence the conclusions drawn from the LCA. It also provides a clearer understanding of the potential variability in the real-world application of MSW-to-biofuel technologies. The results of this sensitivity analysis will help identify the most robust and reliable waste-to-energy conversion technologies, guiding future decisions toward more efficient, low-emission, and high-EROI pathways for climate change mitigation, renewable energy generation, and the circular economy.

### Material flow analysis integration from previous study

The revised MSW management framework presented in Section "Integration with material flow analysis: validation of optimal management strategy" is derived from a previous comprehensive Material Flow Analysis (MFA) study, which systematically evaluated multiple technology combinations and their material transformation pathways across India’s national waste management system^8,93^. That study used STAN 2.7 to model mass balances and identify optimal waste diversion and treatment configurations based on material recovery potential, energy generation capacity, and residue minimization criteria.

In the previous work, various scenarios were analyzed, including: (i) recycling-dominant systems with minimal thermal treatment, (ii) gasification-centred approaches with different feedstock preparation methods, and (iii) integrated multi-stage thermochemical systems^8^. Through systematic comparison of mass balance closure, energy recovery efficiency, and residue generation, the integrated gasification pathway with upstream recyclable segregation emerged as the optimal configuration, achieving 67.5% conversion efficiency to syngas, maximum energy recovery potential (92,711 tpd syngas from 137,351 tpd input), and minimal residual waste generation^8^.

The current LCA study provides complementary environmental validation of this optimal MFA scenario by quantifying its ecological performance across five midpoint impact categories (GWP, SOD, FEP, LU, WC) and comparing it against six alternative MSW management pathways. The convergence of findings, where MIG performs best in both material flow optimization and environmental impact assessment (in the current study), strengthens the evidence base for prioritizing integrated gasification in national MSW policy. The MFA presented in Section "Integration with material flow analysis: validation of optimal management strategy", therefore, serves to: (i) connect the current detailed LCA results to the broader waste system optimization previously conducted, (ii) demonstrate the scalability of the environmentally superior technology to national-level waste flows, and (iii) provide a visual representation of the complete material transformation pathway from waste generation through final product utilization.

## Results and discussions

This section presents the comparative environmental performance of seven waste management scenarios based on five selected midpoint ecological impact categories. Each scenario represents a different combination of WtE technologies, modelled on a per-functional-unit basis. The findings reflect both the direct emissions from treatment processes and the avoided burdens (e.g., from substituted grid electricity) as outlined in the study.

### GWP analysis

**Fig. 4:**
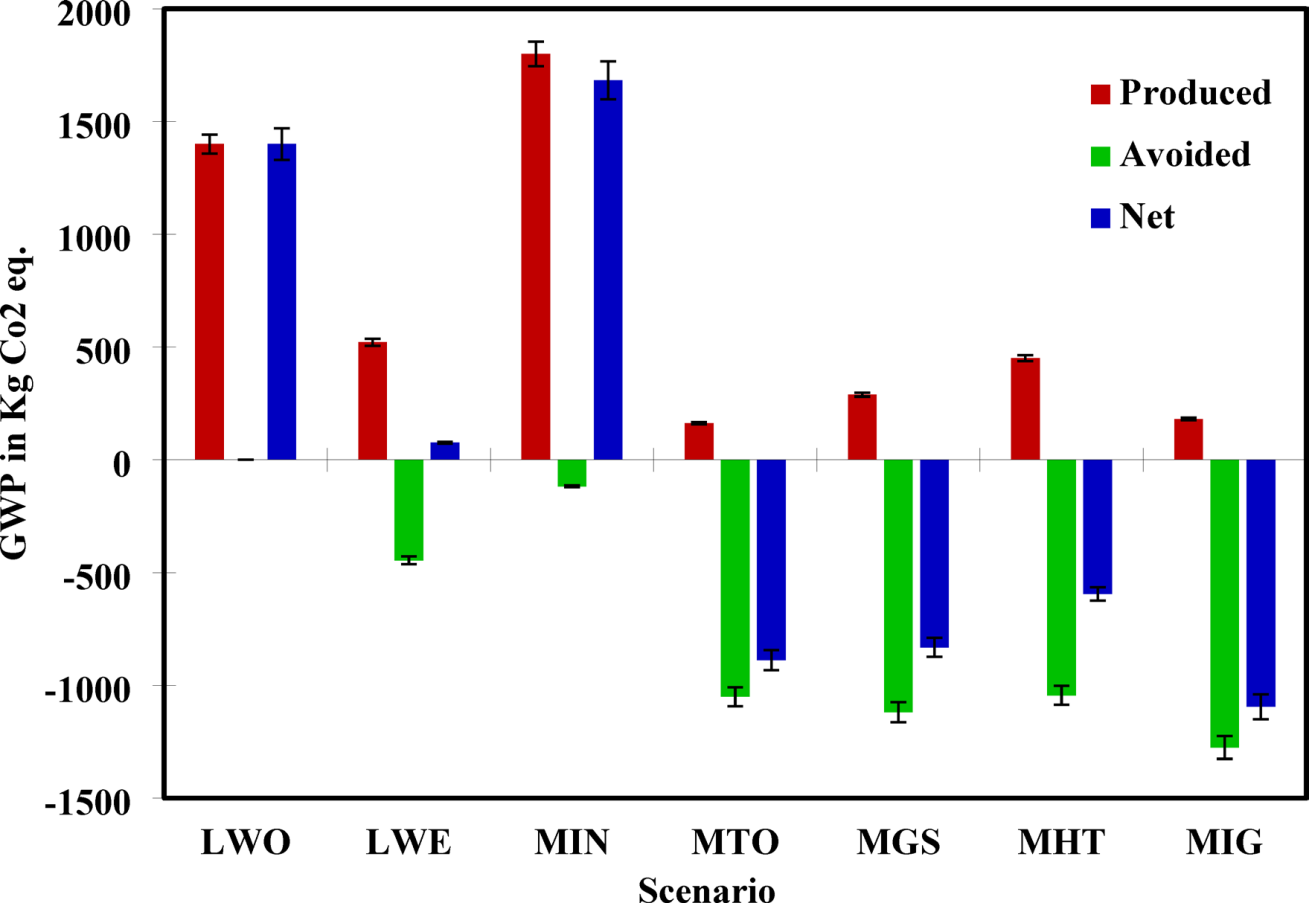
Net GWP for seven MSW management scenarios.

Global Warming Potential (GWP), a critical indicator of climate change impact, varies significantly across the seven modelled scenarios based on the extent of emissions generated and those avoided through energy or material recovery. As seen in the net values, Scenario LWO (S1) had the highest GWP impact at 1400.00 kg CO_2_ eq., reaffirming that open dumping is the most detrimental option for GHG emissions, as shown in Fig. 4. This aligns with the findings of Kumar & Samadder^64^ on landfilling impacts.

Conversely, Scenario MIG (S7), showed the lowest net GWP at − 1095.00 kg CO_2_ eq., indicating a significant environmental benefit. This high performance is attributable to effective energy recovery and the substitution of fossil grid electricity, as previously highlighted in the study of theass incineration scenario by Kumar and Samadder^75^. Scenario MTO (S4) also achieved notable climate benefits, with a net GWP of − 888.08 kg CO_2_ eq., likely due to its optimized mass incineration of combustible fractions (72%), which showed the highest energy recovery in the study area.

Other scenarios, such as MHT (S6) and MGS (S5), delivered moderate climate benefits, with net emissions of − 594.12 and − 831.11 kg CO_2_ eq., respectively, suggesting their viability in contexts where full integration may be constrained. Interestingly, Scenario MIN (S3) yielded a net GWP of 1683.32 kg CO_2_ eq., making it the second-worst option. Scenario LWE (S2) presented a modest improvement over baseline landfilling, with a net GWP of 75.32 kg CO_2_ eq., driven by partial gas recovery and combustion but limited by methane leakage and residual impacts.

### SOD potential analysis

Stratospheric Ozone Depletion (SOD) leads to the emission of long-lived halogenated chemicals such as CFCs and halons, which break down the upper atmospheric ozone. In this study, all scenarios were examined, and it was observed that net SOD values match gross emissions.

**Fig. 5:**
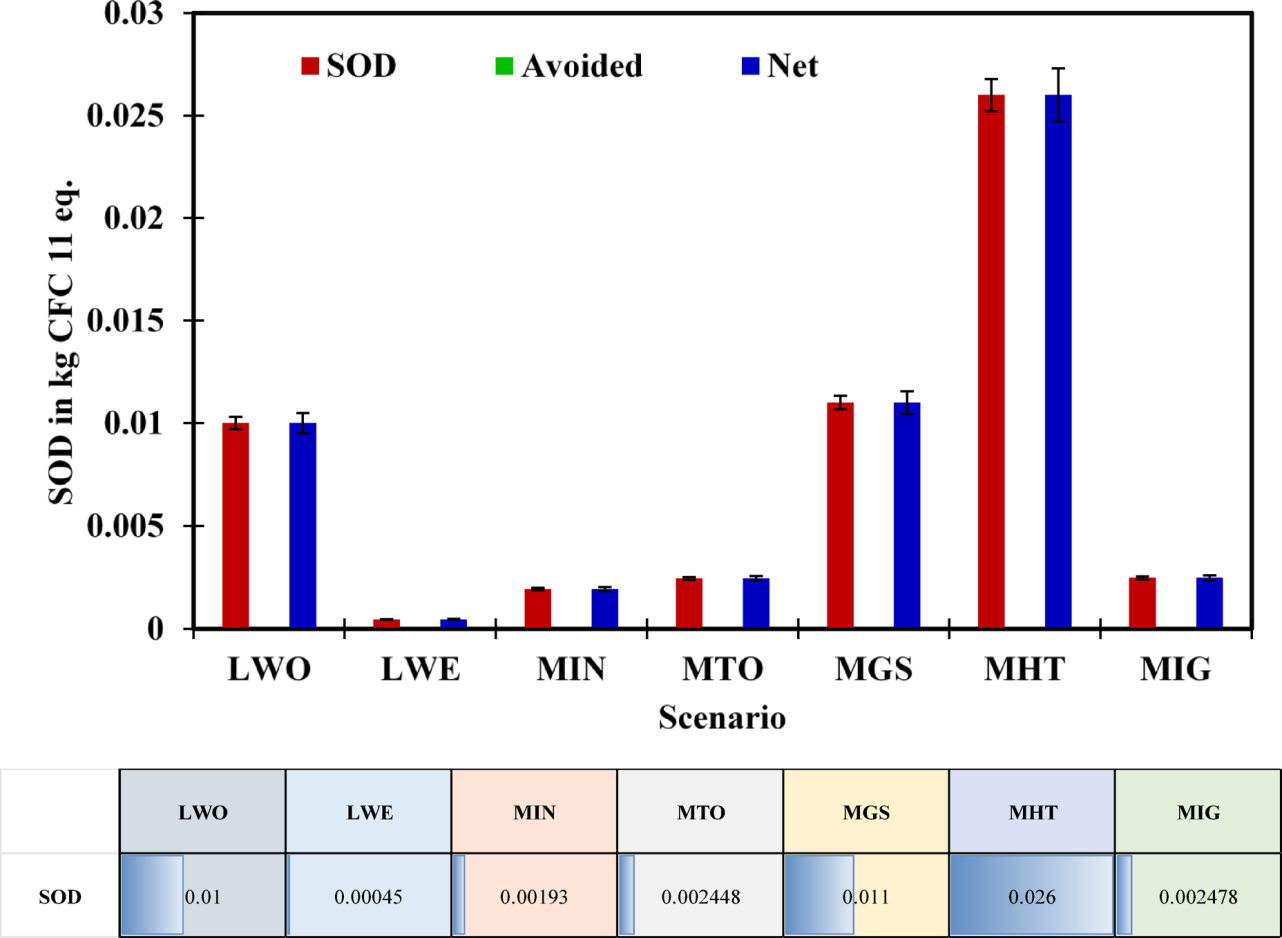
Net SOD potential across MSW management scenarios.

As illustrated in Fig. 5, Scenario LWO (S1) has the most considerable ozone-depleting potential at 0.01 kg CFC-11 eq., possibly due to halogenated organic degradation in unmanaged waste streams. This is similar to prior investigations, which found large SOD loads in landfills without gas capture or flue treatment^63^. The MHT (S6) at 0.026 kg CFC-11 eq. was the worst-performing scenario, suggesting that complex thermal systems and heterogeneous waste fractions may emit trace halogenated pollutants during processing. Scenario MGS (S5) also has a high value of 0.011 kg CFC-11 eq., supporting this pattern. However, Scenario LWE (S2), had the lowest SOD impact at 0.00045 kg CFC-11 eq., showing that methane capture and closed-loop solutions reduce halogen emissions. MTO (S4) and MIG (S7), provided low SOD values (0.0,02,448 and 0.0,02,478 kg CFC-11 eq., respectively), demonstrating that thermal technologies can protect the ozone layer with flue gas treatment systems.

### FEP analysis

**Fig. 6:**
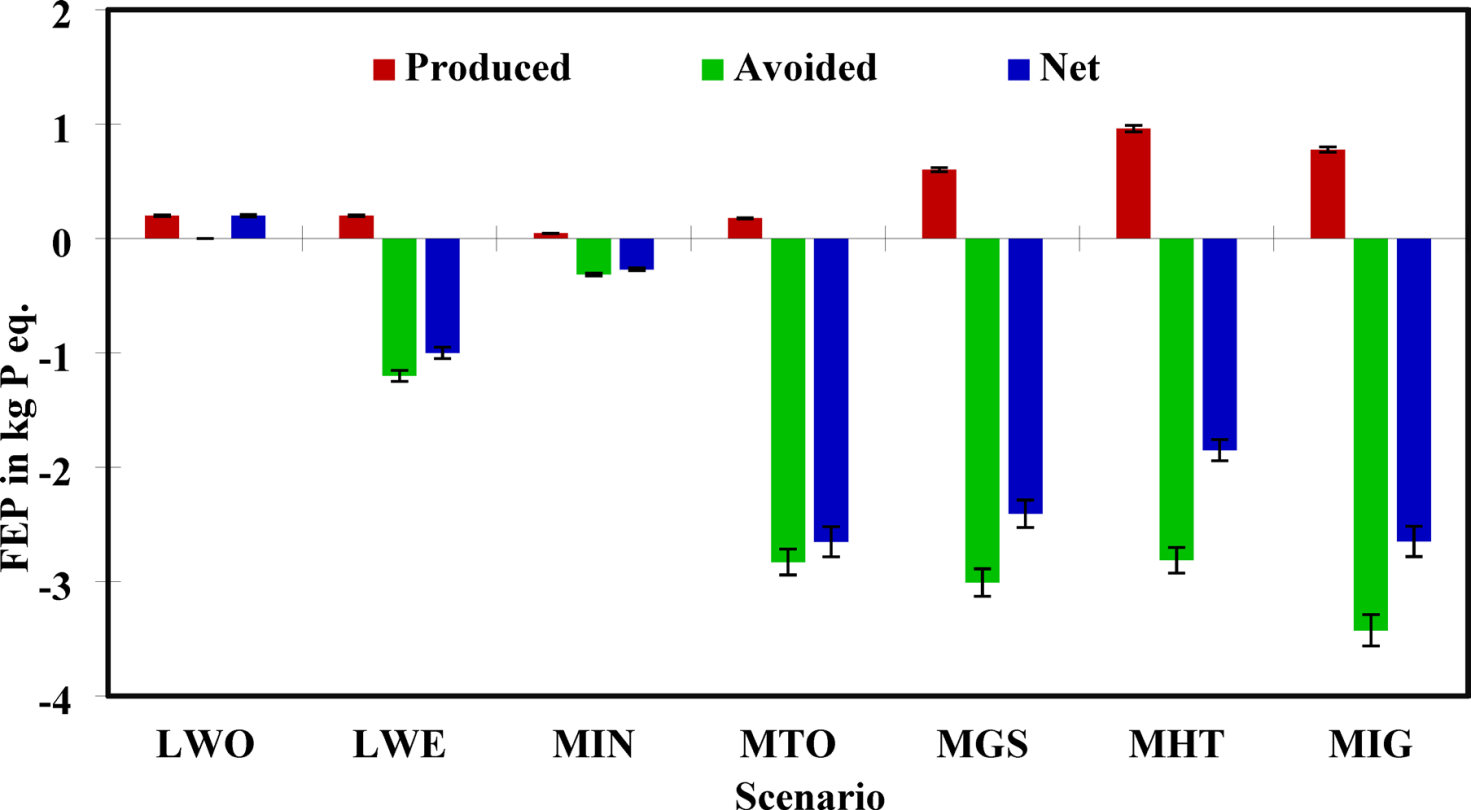
Net FEP for each MSW management scenario.

FEP measures the impact of nutrient discharges, mainly phosphorus, into aquatic ecosystems, leading to algal blooms, oxygen depletion, and ecological imbalance. The net eutrophication values across scenarios varied widely, highlighting the differences in nutrient capture, leachate control, and avoided fertilizer production. The worst-performing scenario in terms of eutrophication was LWO (S1), with a net FEP of 0.20 kg P eq., as shown in Fig. 6 and reported by^94,95^. Unmanaged landfills with no leachate control systems contributed significantly to high nutrient release. Leachates from such systems typically carry ammonia, phosphates, and organic nitrogen, which directly enter water bodies.

In contrast, all other scenarios demonstrated net environmental benefits (negative values), attributed to avoided impacts. Scenario LWE (S2) presented a net FEP of − 0.99964 kg P eq., primarily due to the energy offset and partial methane control. The maximum environmental benefit was observed in Scenario MIG (S7) at − 2.6482 kg P eq., closely followed by MTO (S4) at − 2.6505 kg P eq. These results confirm that combining torrefaction and gasification drastically reduces eutrophic discharges while also avoiding impacts through fertilizer displacement. Notably, MHT (S6) and MGS (S5) also provided substantial savings of − 1.8515 and − 2.4066 kg P eq., respectively. Interestingly, even MIN (S3), which initially had a low gross impact (0.045 kg P eq.), demonstrated a meaningful net reduction of − 0.2685, reinforcing the environmental value of the digestate product.

### LU Potential analysis

LU, expressed in m^2^·a crop equivalents per tonne of MSW, represents the cumulative pressure exerted on land ecosystems due to waste processing, disposal infrastructure, and secondary impacts such as displaced agricultural production. The net LU values indicate the actual spatial burden or benefit of each waste management scenario.

**Fig. 7:**
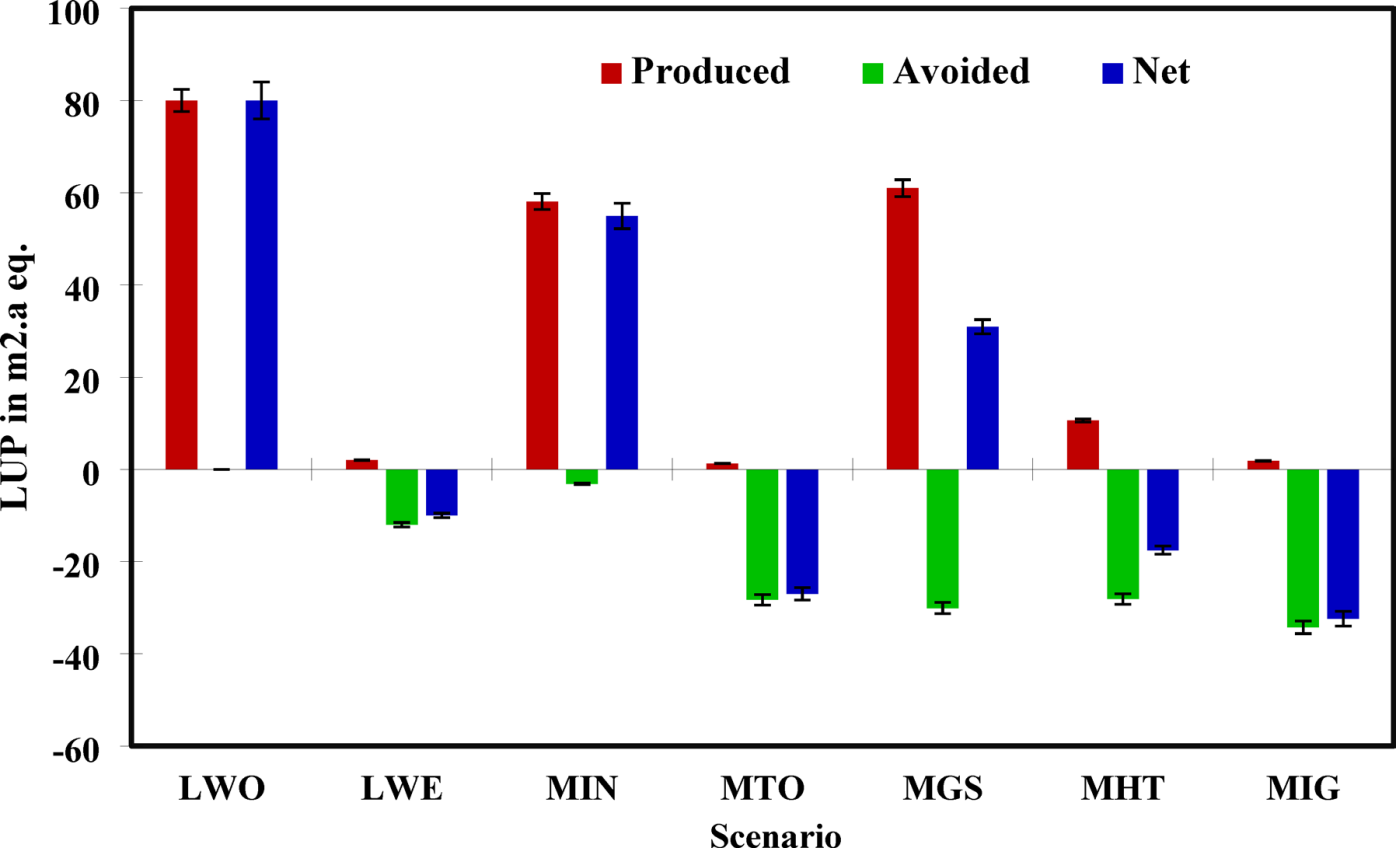
Net LU impact across all scenarios.

The highest net LU impact was observed in Scenario MGS (S5), with a value of 30.924 m^2^·a as shown in Fig. 7. In Scenario MIN (S3), a net LU of 54.955 m^2^·a, confirming that incineration alone, when not coupled with effective landfill diversion, results in substantial land pressure due to waste residues and continued reliance on disposal areas. It is important to note that land use impacts in LCA reflect both direct physical occupation and avoided occupation through energy/material substitution. In coal-dependent grids like India’s, electricity generation displaces substantial upstream land use from mining and ash disposal, meaning high-energy-recovery scenarios can achieve negative net LU even with moderate facility footprints^25,46,90^.

Scenario MIG (S7) (multi-integrated gasification, digestion, and landfilling) achieved the lowest net land use impact at − 32.394 m^2^·a, indicating a strong environmental credit from diverted land occupation, most likely due to minimized residue generation, efficient gasification, and energy substitution. Scenarios MTO (S4) and MHT (S6) also showed very low net land use impacts of − 27.019 m^2^·a and − 17.525 m^2^·a, respectively. These results resonate with Kumar & Samadder^75,92^, who found that mass incineration scenarios consistently demonstrated reduced land dependency due to lower residual volumes. Scenario LWO (S1), the baseline case with conventional open landfilling, showed the highest gross and net land occupation, reflecting long-term spatial burdens from waste accumulation and lack of volume reduction. LWE (S2) offered moderate improvement with a net LU of − 9.9997 m^2^·a, due to avoided electricity production and partial landfill gas recovery, though its residual dependence on landfilling limits its overall benefit.

### WC potential analysis

WC reflects the total freshwater withdrawal required for each treatment process and accounts for avoided consumption through system substitutions. Net values indicate the actual environmental burden or savings in water use. Remarkably, all scenarios except the baseline (LWO) demonstrated net harmful water consumption, suggesting that WTE and integrated recovery systems can effectively reduce overall water burdens through substitution effects.

**Fig. 8:**
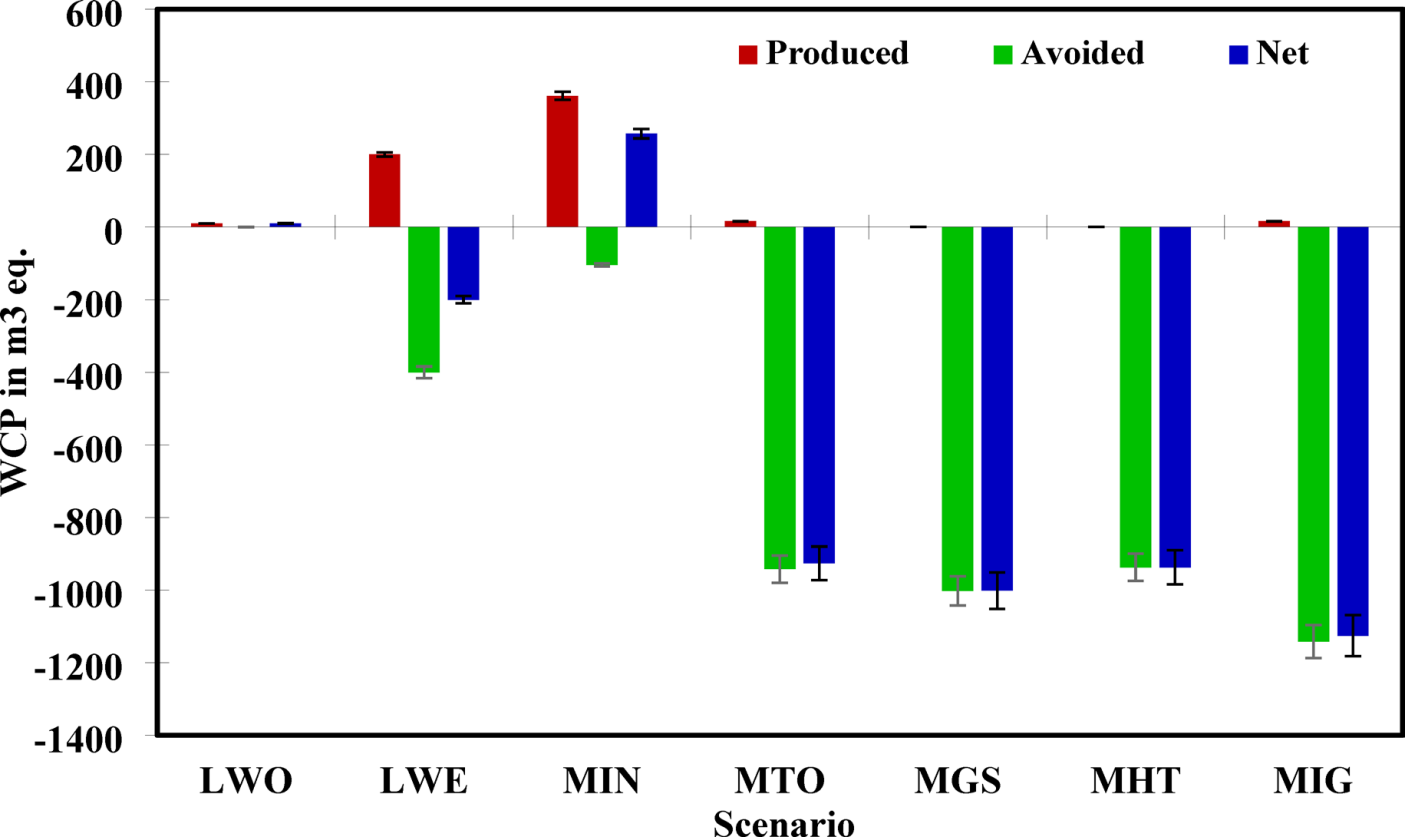
Net WC Potential for each MSW management scenario.

Scenario MIG (S7) emerged as the most water-efficient system, with a net water consumption of − 1125.610 m^3^. This reflects substantial avoided burden, possibly due to a combination of energy production, reduced landfilling, and minimal reliance on water-intensive steps, as shown in Fig. 8. Similarly, MGS (S5) and MHT (S6) also showed high water savings (− 1001.695 m^3^ and − 937.276 m^3^, respectively), thanks to low process water demand and high substitution credit for grid electricity and fertilizers. Scenario MTO (S4) also achieved a substantial net water reduction of − 926.420 m^3^, confirming the efficiency of advanced incineration systems with proper energy recovery and pollution control, as also supported by Kumar & Samadder^75^, who found that water use in incineration was moderate and effectively offset. On the other hand, Scenario MIN (S3), which involves anaerobic digestion, recorded a net water consumption of 257.120 m^3^, the highest among all recovery-oriented scenarios, mainly due to heating requirements in the reactor. Scenario LWE (S2) had a net water use of − 199.997 m^3^, showing some benefit over the baseline. Scenario LWO (S1), however, remains a burden, with no avoided impact and a net WC of 10 m^3^, underlining its unsustainable nature.

A consolidated summary of net environmental impacts across all five midpoint categories and seven scenarios is provided in Table 4, offering a comprehensive quantitative reference for comparative assessment.

**Table 4:** Net environmental impacts across five midpoint categories for all seven MSW management scenarios (per tonne MSW).

Scenario	GWP (kg CO_2_ eq.)	SOD (kg CFC-11 eq.)	FEP (kg P eq.)	LU (m^2^·a)	WC (m^3^)
LWO (S1)	1400.00	0.0100	0.20	68.00	10.00
LWE (S2)	75.32	0.0005	− 1.00	− 10.00	− 200.00
MIN (S3)	1683.32	0.0035	− 0.27	54.96	257.12
MTO (S4)	− 888.08	0.0024	− 2.65	− 27.02	− 926.42
MGS (S5)	− 831.11	0.0110	− 2.41	30.92	− 1001.70
MHT (S6)	− 594.12	0.0260	− 1.85	− 17.53	− 937.28
MIG (S7)	− 1095.00	0.0025	− 2.65	− 32.39	− 1125.61

Values represent net impacts calculated as produced burdens minus avoided burdens through system expansion. Negative values indicate net environmental benefits where avoided impacts exceed effects produced.

### Energy recovery and fossil fuel displacement potential

MSW holds considerable potential as a substitute for fossil fuels through thermochemical recovery processes. The avoided emissions in categories such as GWP and WC reflect the degree to which these scenarios can displace grid electricity generated from coal and reduce freshwater demands associated with thermal power plants. Among the evaluated options, MIG demonstrated the highest fossil fuel substitution potential, with avoided GWP of − 1275.00 kg CO_2_ eq. and water savings of − 1141.750 m^3^/. This scenario integrates multiple treatment methods, maximizing energy output and minimizing residue, thus offering the most comprehensive offset of fossil fuel use. MTO also delivered a strong performance with − 1050.084 kg CO_2_ eq. avoided and − 942.500 m^3^ water conserved, indicating that even partial thermal upgrading of MSW into solid biofuels can significantly reduce reliance on conventional energy sources.

MGS and MHT followed closely, with avoided emissions of − 1119.11 kg CO_2_ eq. and − 1044.12 kg CO_2_ eq., respectively. These technologies convert organic waste into syngas or bio-crude, which can be used directly for power generation or refined into transport fuels, effectively displacing fossil fuels. In contrast, MIN provided environmentally favourable outcomes in some categories, showing relatively low avoided GWP (− 116.68 kg CO_2_ eq.) and modest water savings (− 104.50 m^3^) due to lower energy yields and continued GHG emissions. LWE offered intermediate displacement benefits but was still constrained by methane leakage and limited energy conversion efficiency.

### Integration with material flow analysis: validation of optimal management strategy

Building on the LCA results where the MIG scenario emerged as the most environmentally favourable option across multiple impact categories, achieving an avoided GWP of − 1095.00 kg CO_2_ eq. per tonne MSW, maximum water savings of − 1125.61 m^3^ per tonne, and optimal land use performance (− 32.39 m^2^·a per tonne), this section integrates the current findings with our previous comprehensive Material Flow Analysis^8^. In that prior study, multiple MSW management scenarios combining different technology pathways were systematically evaluated using mass balance modelling in STAN, and the integrated gasification approach with upstream recyclable segregation was identified as optimal based on material recovery efficiency, energy generation potential, and residue minimization. The current LCA provides independent environmental validation of this MFA-optimized scenario, demonstrating that the pathway achieving the best material flow performance also delivers superior environmental outcomes across climate impact, resource consumption, and land-use categories. This convergence of evidence from complementary analytical frameworks, material flow optimization, and LCA, strengthens the technical foundation for future work.

**Fig. 9:**
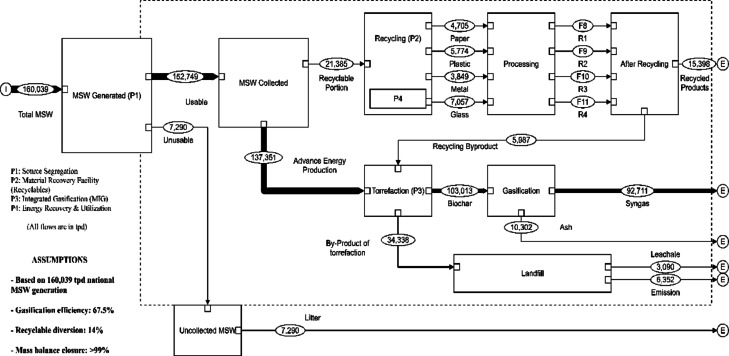
MFA of the optimal MSW management system integrating source segregation and integrated gasification (MIG).

Figure 9 reproduces the optimal material flow configuration from our previous study, showing the theoretical pathways under nationwide adoption of source segregation and centralized MIG processing^8^. India’s MSW generation of 160,039 tonnes per day, with 152,749 tpd collected^8,93^, is managed through a two-stream approach: 21,385 tpd (14% of collected waste) of high-quality recyclables are diverted to material recovery facilities based on existing municipal sorting infrastructure capacity. In contrast, the remaining 137,351 tpd of mixed MSW is processed via integrated gasification. The MFA model, validated with > 99% mass balance closure, indicates that this configuration can theoretically yield approximately 92,711 tpd of syngas with composition suitable for diverse energy applications^4,8^.

This representation emphasizes MIG’s potential to substantially reduce landfill dependency while achieving high levels of energy recovery and emissions reduction, as evidenced by the LCA. When combined with source-level recycling, the proposed pathway maximizes resource utilization while minimizing environmental burdens, including GHG emissions, land use, and freshwater consumption. Though conceptual, this revised model illustrates a replicable and scalable waste-to-energy strategy tailored to the needs of rapidly urbanizing regions.

### Sensitivity analysis

A sensitivity analysis was conducted to assess the robustness of the environmental performance of the best-performing scenario, MIG. The study examined the influence of ± 10% variation in three critical parameters: total MSW input, energy recovery efficiency, and the grid electricity emission factor used in avoided burden calculations.

When the quantity of MSW processed through MIG was increased by 10%, the net GWP increased from − 1095.00 to − 1204.50 kg CO_2_ eq. Conversely, a 10% reduction in waste input reduced the GWP to − 983.20 kg CO_2_ eq., yielding a deviation of approximately ± 5.8%. Similarly, net WC ranged from − 1235.17 to − 1012.20 m^3^, indicating proportional sensitivity to input mass. Adjusting the energy recovery efficiency of the gasification system by ± 10% led to a variation in GWP from − 1147.50 to − 1402.50 kg CO_2_ eq. (± 7.40%). The emission factor for displaced grid electricity by ± 10% (from the baseline) produced avoided GWP values ranging from − 1170.50 to − 1379.00 kg CO_2_ eq., indicating a sensitivity of ± 6.30%. Across all parameters, the scenario maintained its superior environmental performance, demonstrated resilience to input uncertainties, and supported its feasibility as a sustainable large-scale waste management strategy.

This study employed deterministic sensitivity analysis using Excel-based LCA calculations with ReCiPe 2016 characterization factors, prioritizing transparency and traceability in computational procedures^39,40^. While Monte Carlo Simulation provides probabilistic uncertainty quantification, it requires detailed probability distributions for input parameters, which are often unavailable for emerging technologies in developing contexts^21,96^. For instance, gasification efficiency data for Indian MSW compositions are limited to pilot-scale studies with insufficient sample sizes (n = 3–10) to establish robust distributions^24,69^. The ± 10% deterministic range employed here encompasses typical operational variability documented in comparative waste LCA and is appropriate for strategic-level technology prioritization^7,20,29^. Lo Piano and Benini^42^ demonstrate that deterministic sensitivity provides sufficient rigor for comparative assessments where relative ranking is more critical than absolute values. Future facility-specific studies should incorporate probabilistic methods once multi-year operational data from commercial-scale installations becomes available.

## Conclusion

This study presents a scenario-based LCA of seven MSW management pathways, incorporating both conventional disposal methods and advanced waste-to-energy technologies. Among these, the traditional LWO (Scenario 1) exhibited the highest environmental burdens across all five evaluated midpoint impact categories. Even the improved LWE (Scenario 2) showed only moderate ecological benefits due to methane leakage and limited energy recovery.

The thermochemical conversion pathways, such as incineration, torrefaction, gasification, and HTC, demonstrated progressively improved environmental profiles. However, Scenario 7 (MIG) emerged as the most environmentally favourable option, offering the lowest net GWP (− 1095.00 kg CO_2_ eq.), the maximum water savings (− 1125.61 m^3^), and the minimum land-use impact (− 32.394 m^2^·a). These benefits are primarily due to efficient energy recovery, reduced residuals, and a high substitution potential for fossil-based electricity and fertilizers.

While MIG demonstrated superior environmental performance across all evaluated impact categories, its practical implementation in the Indian context faces significant techno-economic challenges that warrant consideration. Capital costs for integrated gasification facilities are substantially higher (estimated at 2–3 × those of conventional incineration), requiring strong policy support mechanisms such as enhanced tipping fees, renewable energy incentives, or carbon credit revenues under emerging trading schemes. Additionally, MIG demands higher feedstock quality specifications (pre-sorted, moisture content < 25%) compared to landfilling, necessitating investment in upstream waste segregation and mechanical–biological treatment infrastructure. The technology also requires skilled operators and continuous process monitoring, contrasting with simpler conventional disposal methods. Despite these barriers, declining technology costs (gasification CAPEX reduced ~ 30% during 2015–2023) and supportive policy frameworks (SWM Rules 2016 mandating energy recovery for cities > 1 million) create favourable conditions for phased deployment. The substantial net environmental benefits quantified in this study (− 1095.00 kg CO_2_ eq./tonne, − 1126.00 m^3^ water savings/tonne) provide a strong rationale for prioritizing MIG in national waste management strategies, particularly through pilot projects in metropolitan areas with established waste segregation systems before nationwide scale-up. Future studies should integrate techno-economic analysis with LCA results to provide comprehensive decision support for technology adoption.

The MFA further confirmed MIG’s technical viability by modelling a revised urban MSW system that treats over 137,000 tonnes of waste daily, yielding approximately 92,700 tonnes of syngas for energy and fuel applications. Sensitivity analysis confirmed MIG’s environmental superiority remains robust under ± 10% parameter variations. While deterministic sensitivity is appropriate for comparative strategic assessment, future facility-specific studies should incorporate Monte Carlo Simulation when sufficient operational data becomes available. These findings emphasize the need for a systemic shift away from landfilling and toward integrated, high-efficiency thermochemical processes, particularly integrated gasification, to enable circular and sustainable waste management in urban environments.

## Data Availability

“The data that support the findings of this study are available from the corresponding author upon reasonable request. All data used in the analysis have been obtained from publicly accessible sources, institutional records, or were generated during this research.”

## Abbreviations

AD: Anaerobic digestion
ASTM: American society for testing and materials
CFC-11 eq.: Trichlorofluoromethane equivalent
CH_4_: Methane
CHP: Combined heat and power
CO_2_: Carbon dioxide
EF: Emission factor
EROI: Energy return on investment
FE: Freshwater eutrophication
FEP: Freshwater eutrophication potential
GHG: Greenhouse gas
GWP: Global warming potential
HHV: Higher heating value
HTC: Hydrothermal carbonization
ISO: International organization for Standardization
LCA: Life cycle assessment
LCI: Life cycle inventory
LCIA: Life cycle impact assessment
LHV: Lower heating value
LU: Land use
LWE: Landfill with energy recovery
LWO: Landfill without energy recovery
MFA: Material flow analysis
MGS: MSW gasification
MHT: MSW hydrothermal carbonization
MIN: MSW incineration
MIG: MSW integrated gasification
MTO: MSW torrefaction
MSW: Municipal solid waste
NH_3_: Ammonia
NO_x_: Nitrogen oxides
O_2_: Oxygen
PM: Particulate matter
RDF: Refuse-derived fuel
SDGs: Sustainable development goals
SOD: Stratospheric ozone depletion
SO_2_: Sulfur dioxide
TPD: Tonnes per day
WC: Water consumption
WtE: Waste-to-energy
